# Nicotine exacerbates liver damage in a mice model of Ehrlich ascites carcinoma through shifting SOD/NF-κB/caspase-3 pathways: ameliorating role of *Chlorella vulgaris*

**DOI:** 10.1007/s00210-024-03120-9

**Published:** 2024-05-09

**Authors:** Ehsan H. Abu-Zeid, Eman W. El-Hady, Gehan A. Ahmed, Yasmina M. Abd-Elhakim, Doaa Ibrahim, Noura A. Abd-Allah, Ahmed H. Arisha, Mohammed S. Sobh, Azza M. A. Abo-Elmaaty

**Affiliations:** 1 Department of Forensic Medicine and Toxicology, Faculty of Veterinary Medicine, Zagazig University, Zagazig, 44519 Egypt; 2 Department of Forensic Medicine and Clinical Toxicology, Faculty of Medicine, Zagazig University, Zagazig, 44519 Egypt; 3 Department of Nutrition and Clinical Nutrition, Faculty of Veterinary Medicine, Zagazig University, Zagazig, 44519 Egypt; 4 Department of Clinical Pathology, Faculty of Veterinary Medicine, Zagazig University, Zagazig, 44519 Egypt; 5 Department of Animal Physiology and Biochemistry, Faculty of Veterinary Medicine, Badr University in Cairo (BUC), Badr City, Cairo Egypt; 6 Department of Physiology, Faculty of Veterinary Medicine, Zagazig University, Zagazig, 44519 Egypt; 7 Department of Pathology, Faculty of Veterinary Medicine, Zagazig University, Zagazig, 44519 Egypt; 8 Department of Pharmacology, Faculty of Veterinary Medicine, Zagazig University, Zagazig, 44519 Egypt

**Keywords:** Nicotine, *Chlorella vulgaris*, Hepatotoxicity, Ehrlich’s ascites carcinoma, Nuclear Factor-kappa β *NF-κB*, Oxidative stress

## Abstract

Nicotine, a pervasive global environmental pollutant, is released throughout every phase of the tobacco’s life cycle. This study examined the probable ameliorative role of *Chlorella vulgaris* (ChV) extract against nicotine (NIC)-induced hepatic injury in Ehrlich ascites carcinoma (EAC) bearing female Swiss mice. Sixty female Swiss mice were assigned to four equal groups orally gavaged 2% saccharin 0.2 mL/mouse (control group), orally intubated 100 mg ChV /kg (ChV group), orally intubated 100 µg/mL NIC in 2% saccharin (NIC group), and orally intubated NIC + ChV as in group 3 and 2 (NIC+ChV group). The dosing was daily for 4 weeks. Mice from all experimental groups were then inoculated intraperitoneally with viable tumor cells 2.5 × 10^6^ (0.2 mL/mouse) in the fourth week, and the treatments were extended for another 2 weeks. The results have shown that NIC exposure significantly altered the serum levels of liver function indices, lipid profile, LDH, and ALP in the NIC-exposed group. NIC administration significantly increased hepatic inflammation, lipid peroxidation, and DNA damage-related biomarkers but reduced antioxidant enzyme activities. NIC exposure downregulated *SOD1*, *SOD2*, *CAT*, *GPX1*, and *GPX2* but upregulated *NF-κB* hepatic gene expression. Notably, the presence of the EAC cells outside the liver was common in all mice groups. Liver tissue of the NIC-exposed group showed multifocal expansion of hepatic sinusoids by neoplastic cells. However, with no evidence of considerable infiltration of EAC cells inside the sinusoids or in periportal areas in the NIC + ChV groups. NIC significantly altered caspase-3, Bax, and BcL2 hepatic immune expression. Interestingly, ChV administration significantly mitigates NIC-induced alterations in hepatic function indices, lipid profile, and the mRNA expression of antioxidant and* NF-κB* genes and regulates the caspase-3, Bax, and BcL2 immunostaining. Finally, the in vivo protective outcomes of ChV against NIC-induced hepatic injury combined with EAC in female Swiss mice could suggest their helpful role for cancer patients who are directly or indirectly exposed to NIC daily.

## Introduction

Environmental risk factors, such as exposure to pollutants, contribute to the initiation, progression, and increase of the cancer severity (Esterhuizen et al. 2023). In terms of global public health, tobacco smoke is now at the top of the list of hazardous environmental pollutants (Zhou 2019). According to the Centers for Disease Control and Prevention, smoking is the greatest cause of mortality in Egypt and the United States, with more than 34,000 and 480,000 deaths each year, respectively (Abdel-Hady and El-Gilany 2020). Persistent tobacco smoking in cancer patients has several negative outcomes, including treatment failure, worse quality of life, lower survival rates, and an increased chance of second primary tumors (Warren et al. 2014; Jassem 2019). Aside from cancer type, stage, and site, persistent smoking is regarded as the best negative predictor of survival in cancer patients (Jassem 2019). The effects of nicotine (NIC) and cigarette smoke on several organ systems’ stem cell-associated pathways that are important for carcinogenesis are becoming more clear (Thong et al. 2019).

NIC is a major alkaloid that accounts for about 95% of the total alkaloid content in commercial tobacco (Nwosu and Krasowski 2023). Various stages of the tobacco life cycle contribute to environmental contamination with NIC. These stages include tobacco cultivation, cigarette production, combustion of cigarettes, and the release of NIC and its metabolites into human wastewater streams (Beutel et al. 2021). The toxic effects of NIC are most pronounced in the liver since it is responsible for most of NIC’s metabolism and biotransformation (Ateyya et al. 2017). Chronic NIC administration promotes activation of cytochrome P-450 and increased reactive oxygen species (ROS) generation (Yue et al. 2009). NIC promotes ROS production by activating the nicotinamide adenine dinucleotide phosphate-oxidase enzyme (NADPH oxidase/NOX1) (Asano et al. 2012). NIC has further been implicated in hepatic oxidative stress, apoptosis, and lipid dysmetabolism (Chen et al. 2018; Hasan et al. 2019; Dangana et al. 2020). NIC exposure resulted in hepatic tissue inflammation chiefly via augmented expression of NADPH oxidase enzyme, inducible nitric oxide synthase (iNOS), nuclear factor kappa B (NF-κB), and tumor necrosis factor-alpha (TNF-α) (Khaled et al. 2020). Thus, based on the information obtained from these studies, we have designed this experiment to appraise the impact of NIC exposure on cancer progression and liver function in an in vivo mice model bearing Ehrlich ascites carcinoma (EAC). Because of its similarities to human tumors, EAC is often used to assess the anticancer activity of different drugs or natural products using its ascetic forms (Hashem et al. 2020; Oraby et al. 2023).

Research on microalgae has garnered significant interest because of the potential health benefits they may offer (Lozoya-Pérez et al. 2024). *Chlorella vulgaris* (ChV) is a single-celled microalgae that grows in fresh water and is considered a safe dietary supplement by the United States Food and Drug Administration (USFDA) (Silva et al. 2019; Elif 2023). ChV is a high-nutrient dietary source comprising 61.6% proteins, 12.5% fat, and 13.7% carbs and consists of more than 20 minerals and vitamins (Perveen et al. 2022). Furthermore, ChV contains numerous antioxidants such as chlorophyll, carotenoids, tocopherol, ascorbic acid, omega-6, and omega-3 polyunsaturated fatty acids, polysaccharides, essential amino acids, α- and β-carotene, and vitamins (Panahi et al. 2016). In ChV, alpha and β-carotene interact with different ROS and consequently suppress the oxidation processes in cellular and lipid compartments (Nass et al. 2022). Besides, chlorophylls found in ChV aid in lowering oxidative DNA damage and lipid peroxidation (LPO) through decreasing chelating ROS and metal ions (Queiroz et al. 2011). According to a study compromising 32 different microalgae species, ChV has great antioxidant activity (Goiris et al. 2012). ChV has been shown to provide multiple potential benefits to human health such as antidiabetic (Ebrahimi-Mameghani et al. 2017), hypo-cholesterolemic (Sherafati et al. 2022), cardio-protective (Barghchi et al. 2023), and antiparasitic (Melo et al. 2024). Besides, ChV has been shown to possess antioxidant, anti-inflammatory, and antipapoptotic activities (Abdel-Aziem et al. 2018; Mohamed et al. 2023). In patients with non-alcoholic fatty liver disease, ChV has been suggested as an adjunctive therapy to improve liver function (Ebrahimi-Mameghani et al. 2017). ChV regulated antioxidant enzyme activity and malondialdehyde (MDA) levels and decreased tumor numbers in ethionine-induced liver cancer in rats (Sulaiman et al. 2006). Supplementation with ChV for 6 weeks in Iranian chronic cigarette smokers significantly improved antioxidant status and reduced LPO (Panahi et al. 2013). ChV also exhibited its antioxidant competencies in the liver in numerous modes: from suppression of ROS release to augmentation of antioxidant enzymes (superoxide dismutase (SOD) and catalase (CAT)) activities and upregulation of main antioxidant genes (Sikiru et al. 2019). Furthermore, ChV supplementation in carbon tetra chloride (CCL_4_)-exposed rats slows liver fibrosis development by blocking the TGF signaling pathway (Mohseni et al. 2021).

Although many studies on ChV have been undertaken, there is still insufficient knowledge concerning the hepatoprotective effect of ChV against NIC. Therefore, the present trial aimed to evaluate the possible ameliorating role of ChV against hepatotoxicity induced by NIC oral intoxication in female Swiss mice combined with EAC induction through biochemical, molecular, histopathological, and immunohistochemical analyses.

## Material and methods

### Tested compounds

NIC and saccharin were provided from Sigma-Aldrich Chemie GmbH and (CAS Name: 54–11-5; 81–07-2) and product numbers N3876-5ML and 109,185, respectively. The ChV ethanolic extract 80% used in this investigation was a kind gift of Dr. Mohamed A.A.R., Department of Forensic Medicine and Toxicology, Faculty of Veterinary Medicine, Zagazig University, which was prepared according to Elsawi et al. (2018), and evaluated using HPLC (Agilent 1100, Merck KGaA, Darmstadt, Germany). The extract contains numerous flavonoids, phenolic, and polysaccharide compounds, including quercetin, rutin, hesperidin, 7-OH flavone, ellagic, cinnamic, glucuronic acid, catechol, and Rhamnose (Mohamed et al. 2022). The EAC parent line was graciously given by Egypt’s National Cancer Institute at Cairo University. The viability of the parent EAC line was investigated according to Scheid et al. (1972). EAC was maintained in Swiss mice by serialized intraperitoneal transplantations of EAC 2.5 × 10^6^ tumor cells/0.2 mL in female Swiss albino mice.

### Animals and experimental design

A total of sixty Swiss female mice (24 ± 2 g average body weight) were provided from the veterinary medicine laboratory animal farm at Zagazig University (El-Sharkia, Egypt). Mice complied with conventional laboratory hygiene settings, including a temperature range of 22–28 °C, a 12-h light/dark cycle, and a relative humidity range of 50–60%. The animals were accustomed to the laboratory conditions for 2 weeks before the trial, freely allowed to obtain water and balanced feed during the acclimatization and experimental periods. The experimental measures were approved by the Institutional Animal Care and Use Committee (IACUC) of the Faculty of Veterinary Medicine, Zagazig University, Egypt (ZU-IACUC/2f/ 278/2022). Mice were separated into four groups (*n* = 15): the first control group (C, orally received 2% saccharin dissolved in distilled water 0.2 mL/mouse), the second ChV group (ChV, orally intubated 100 mg/kg ChV BW dissolved in distilled water according to Justo et al. (2001), the third NIC group (NIC, were orally gavaged with 100 µg/ml NIC in 2% saccharin according to Sparks and Pauly (1999), the fourth NIC + ChV group (orally co-administered NIC and ChV by the same doses and routes in groups 3 and 2). The dosing was orally and daily for the whole experimental period of 4 weeks using a bent stainless steel stomach tube. Then, mice from all experimental groups were inoculated intraperitoneally (i.p) with 2.5 × 10^6^ viable tumor cells in 0.2 mL per mouse. The Trypan blue dye exclusion method was used to determine viability, which was always determined to be at least 95%. Then, mice were dosed daily for another 2 weeks.

### Sampling

Blood samples were taken from the retro-orbital plexus of each mouse from all experimental groups. Blood was collected in a plain tube, left for coagulation, and centrifuged at 3000 rpm for 10 min. The serum samples were kept at − 20 °C for the biochemical analysis of hepatic enzymes and lipid profile. Additionally, for the determination of hepatic mRNA expression levels of inflammatory and oxidative stress-related genes, a small portion of liver tissues (30 mg) was dissected from the euthanized mice from all experimental groups and was immersed in about five volumes of RNAlater® solution. The samples were stored at − 80 °C for following RT-PCR procedures. For the assessment of oxidative stress and inflammatory-related biomarkers, hepatic tissue (0.5 g) was dissected and homogenized (WiseTis HG-15D homogenizer, Daihan Scientific Co., Seoul, Korea). Finally, liver specimens were immediately post-fixed in 10% neutral-buffered formalin for histopathological and immunohistochemical assessment.

### Biochemical estimations of serum levels of lipid profile and hepatic enzyme activities

Triglycerides, total cholesterol, low-density lipoproteins (LDL-C), high-density lipoproteins (HDL-C), total proteins, albumin, alkaline phosphatase (ALP), aspartate aminotransferase (AST), and alanine aminotransferase (ALT) level in the serum were determined by colorimetric kits of Bio-diagnostic CO (Dokki, Giza, Egypt) with the following catalog number: TR 20 30, CH 12 20, CH 12 30, CH 12 31, TP 20 20, AB 10 10, AP 10 21, AS 10 61, and AL 10 31, respectively. Serum globulin level was estimated by deducting the albumin level obtained from the total protein. The Spinreact Co. (Santa Coloma, Spain) lactate dehydrogenase (LDH) kit was used to determine LDH levels. Also, very low-density lipoprotein (VLDL-C) was calculated according to the method of (Friedewald et al. 1972).

### Biochemical assessments of inflammation and oxidative stress-related indices in hepatic tissue

The hepatic levels of reduced glutathione (GSH), nitric oxide (NO), and MDA, together with the activities of glutathione peroxidase (GPX), SOD, and CAT, were measured via using the kit of Bio-diagnostic CO (Dokki, Giza, Egypt) with CAT. No. GR 25 11, NO. 25 33, MD 25 29, GP 2524, SD 25 21, and CA 25 17, respectively. Meanwhile, TNF-α, IL-6, interleukin-1 (IL-1), 8-hydroxy-deoxyguanosine (8-OHdG), and protein carbonyl (PCO) were determined by specific mouse ELISA kits of MyBioSource Co. (San Diego, CA, USA) with Cat. Nos. MBS825075, MBS2508516, MBS036031, MBS700097, and MBS2600846, respectively.

### Real-time quantitative PCR (RT-qPCR) investigation of hepatic oxidative stress and inflammation-related genes

TRIzolTM (Invitrogen; Thermo Fisher Scientific, Inc. Waltham, MA, USA) was used to extract total RNA. cDNA was synthesized using the HiSenScript™ RH (-) cDNA Synthesis Kit (iNtRON Biotechnology Co., South Korea) (Arisha and Moustafa 2019). The RT-PCR was accomplished in an Mx3005P Real-Time PCR System (Agilent Stratagene, USA) by TOPreal™ qPCR 2X PreMIX (SYBR Green with low ROX) following the manufacturer’s guidelines. An initial denaturation at 95 °C for 15 min was followed by 40 cycles of denaturation for 30 s at 95 °C under the PCR cycling conditions, annealing at 60 °C for 60 s, and extension for 60 s at 72 °C. The oligonucleotide-specific primers are shown in Table 1 in line with Dong et al. (2008), Nguyen et al. (2016), and Ying et al. (2019). The 2^−ΔΔ^CT comparative technique was used to calculate the relative fold changes in gene expression after normalizing the target genes’ expression levels to Gapdh (Livak and Schmittgen 2001).

**Table 1:** Primer sequences used for RT-PCR analysis

Gene	Forward primer (5′–3′)	Reverse primer (5′–3′)	Reference
*GAPDH*	CGTGTTCCTACCCCCAATGA-3	ATGTCATCATACTTGGCAGGTTTCT	**(**Ying et al. 2019**)**
*NF-κB*	CACTGAGGAGACCACCCAAG	GTAAACGCCGAAGATGATGG	**(**Ying et al. 2019**)**
*SOD1*	GTGATTGGGATTGCGCAGTA	TGGTTTGAGGGTAGCAGATGAGT	**(**Dong et al. 2008**)**
*SOD2*	TTAACGCGCAGATCATGCA	GGTGGCGTTGAGATTGTTCA	**(**Dong et al. 2008**)**
*CAT*	TGAGAAGCCTAAGAACGCAATTC	CCCTTCGCAGCCATGTG	**(**Dong et al. 2008**)**
*GPX1*	CACCGAGATGAACGATCTG	CAGGTCGGACGTACTTGAG	**(**Nguyen et al. 2016**)**
*GPX2*	ACCGATCCCAAGCTCATCAT	CAAAGTTCCAGGACACGTCTGA	**(**Dong et al. 2008**)**

*GAPDH*, glyceraldehyde 3-phosphate dehydrogenase; *NF-κB*, nuclear factor kappa B; *SOD1*, superoxide dismutase 1; *SOD2*, superoxide dismutase 2; *CAT*, catalase; *GPX1*, glutathione peroxidase 1; *GPX2*, glutathione peroxidase 2

### Histopathological and immunohistochemical investigations of Bax, caspase-3, and Bcl2

The formalin-fixed liver specimens were rinsed, dehydrated in escalating degrees of ethyl alcohol, clarified in xylene, and further processed for the paraffin technique (Layton et al. 2018). A microtome (Leica RM 2155, England) was used to slice three successive paraffin sections with a thickness of five microns. Before the microscopical examination, the sections were stained with hematoxylin and eosin as per protocol, mounted in DPX, and covered with a glass slide (Suvarna and Layton 2013).

For immunohistochemical workup, the previously obtained tissue, ten slides per biomarker per group (5 µm paraffin sections) were managed for immunohistochemical staining following the ABC technique described by Hsu et al. (1981) using the following primary antibodies: (a) for caspase-3, rabbit monoclonal (EPR18297) to anti-mice caspase-3 (Abcam, Cat. no. ab184787, dilution 1;1000); (b) for Bax, rabbit polyclonal anti-Bax antibody (Abcam, Cat. no. ab53154, dilution 1; 50); (c) for BcL-2, rabbit monoclonal (EP10625) to anti-mice BcL-2 (Abcam, cat. no. ab203516, dilution 1;500). (ABCAM Inc., Cambridge, UK). Also, negative sections from the control were obtained by incubating with phosphate buffer saline to replace the primary antibodies.

To measure positive reactivity, images of different sections stained with antibodies were examined under a microscope powered by an Olympus BX-50 in Tokyo, Japan, with a 1/2 × photo adapter and a 40 × objective. The images were captured using an Olympus LC20 digital camera, which was put on an Olympus microscope. The images were analyzed using a computer with an Intel® Core I3® and the Russian program Video Test Morphology 5.2, which has a dedicated method for immunohistochemical analysis and stain quantification. The system determined caspase-3, Bax, and Bcl2 expression percentages in a certain region. For quantitative analysis, we selected five representative areas in total with both positive cell areas and areas without expression. If a tissue section had areas with both low abundance and high abundance of stained cells, both areas were selected as representative areas and included in the analysis. Individual cells were identified by strong brown stain and Image analysis software (JID801D) assessed positive cells. We counted the number of positive expressed cells per mm^2^ and converted them into area %. The cell counting was repeated three times for each area. All images were analyzed in a blinded fashion.

### Statistical method

The statistical data was analyzed using IBM SPSS, version 21, with a one-way analysis of variance (ANOVA). Tukey’s multiple range test was used for pairwise comparisons between the experimental groups. The data is displayed as the average with or without the standard error. Statistical significance was defined as a probability level lower than 0.05. GraphPad Prism 8 from GraphPad Software Inc. in San Diego, CA, USA, was used to generate every graph.

## Results

### Effect of ChV and/or NIC exposure combined with EAC on liver function and lipid profile

The observed changes in liver function and lipid profile in the serum of EAC-bearing female Swiss mice exposed to ChV and/or NIC are shown in Table 2. Administration of ChV resulted in a significant decrease in ALT, AST, ALP, and LDH levels by 30.13%, 21.73%, 12.07%, and 12.45%, respectively, relative to the C group. Besides, ChV exposure significantly decreased TGs, TC, LDL-C, and VLDL-C by 31.48%, 8.79%, 22.75%, and 13.96%, respectively, but resulted in a non-significant increase in HDL-C by 18.84% than the C group. Moreover, ChV exposure induced a significant increase in total proteins, albumin, and A/G ratio by 15.37%, 27.20%, and 22.13%, respectively, while serum levels of globulins and HDL-C were non-significantly increased by 1.24% and 18.84%, respectively, than the C group.

**Table 2:** Effect of *Chlorella vulgaris* (ChV) and/or nicotine (NIC) exposure on liver function and lipid profile indices in serum of Ehrlich ascites carcinoma (EAC)-bearing female Swiss mice

Parameters	Groups			
	Control	ChV	NIC	NIC + ChV
ALT(U/L)	24.33 ± 0.71	17.00 ± 0.73 ^*^	47.00 ± 1.63 ^*^	30.00 ± 1.07 ^* #^
AST (U/L)	38.33 ± 0.84	30.00 ± 0.86 ^*^	51.50 ± 1.05 ^*^	44.83 ± 1.66 ^* #^
ALP (IU/L)	58.00 ± 1.59	51.00 ± 0.76 ^*^	87.00 ± 1.69 ^*^	70.33 ± 0.84 ^* #^
LDH (U/L)	362.83 ± 7.53	317.66 ± 8.48 ^*^	503.66 ± 12.47 ^*^	420.33 ± 10.81 ^#^
Total proteins (g/dl)	7.09 ± 0.06	8.18 ± 0.22 ^*^	4.92 ± 0.07 ^*^	6.46 ± 0.22 ^#^
Albumin (g/dl)	3.86 ± 0.17	4.91 ± 0.19 ^*^	2.52 ± 0.02 ^*^	3.55 ± 0.16 ^#^
Globulins (g/dl)	3.23 ± 0.14	3.27 ± 0.02	2.40 ± 0.07 ^*^	2.91 ± 0.08 ^#^
A/G ratio	1.22 ± 0.09	1.49 ± 0.05 ^*^	1.10 ± 0.06	1.22 ± 0.05
Cholesterol (mg/dl)	170.66 ± 2.23	155.66 ± 2.43^*^	243.33 ± 2.13 ^*^	192.66 ± 2.69^*#^
Triglycerides (mg/dl)	126.00 ± 1.67	111.00 ± 2.98 ^*^	172.00 ± 4.57 ^*^	145.33 ± 2.07^* #^
HDL-C (mg/dl)	51.33 ± 1.05	61.00 ± 4.11	27.00 ± 2.22 ^*^	37.33 ± 1.28^* #^
LDL-C (mg/dl)	95.96 ± 1.84	74.13 ± 3.76 ^*^	181.93 ± 2.49 ^*^	126.26 ± 3.39^* #^
VLDL-C (mg/dl)	23.86 ± 0.62	20.53 ± 0.36 ^*^	34.40 ± 0.91^*^	29.06 ± 0.41^* #^

*ALT*, alanine aminotransferase; *AST*, aspartate aminotransferase; *ALP*, alkaline phosphatase; *LDH*, lactate dehydrogenase; *A/G ratio*, albumin/globulin ratio; *HDL-C*, high-density lipoproteins; *LDL-C*, low-density lipoproteins; *VLDL-C*, very low-density lipoproteins. Values are mean ± SEM of six mice per experimental group. *P < 0.05 vs control, ^#^*P* < 0.05 vs NIC

NIC exposure significantly increased ALT, AST, ALP, and LDH serum levels by 93.18%, 34.36%, 50%, and 38.81%, respectively, but significantly reduced the serum content of total proteins, albumin, and globulins by 30.75%, 34.97%, and 25.70%, respectively, while A/G ratio was non-significantly decreased by 9.84% when matched with the C group. Also, NIC exposure significantly increased serum levels of TG, TC, VLDL-C, and LDL-C by 6.17%, 42.58%, 44.17%, and 89.59%, respectively, and significantly decreased HDL-C serum levels by 47.40% than the C group.

ChV oral dosing in the NIC + ChV group significantly reestablished the increased ALT, AST, and ALP serum levels by 23.30%, 16.96%, and 21.26%, respectively, while LDH serum levels non-significantly restored by 15.85% than the C group. Additionally, ChV administration non-significantly regenerated the NIC-induced decrease of serum levels of total protein, albumin, and globulin to (8.89%, 8.03%, and 9.91% decrease) compared with the C group. Also, ChV dosing in the NIC + ChV group significantly restored the increased serum levels of TG, TC, LDL-C, and VLDL-C to 10.29%, 12.89%, 31.58%, and 21.79%, respectively, and significantly reinstated the NIC-induced decrease in HDL-C serum levels to 27.27% than the C group.

### Effect of ChV and/or NIC exposure combined with EAC on inflammatory and oxidative stress-associated indices in hepatic tissue

**Fig. 1:**
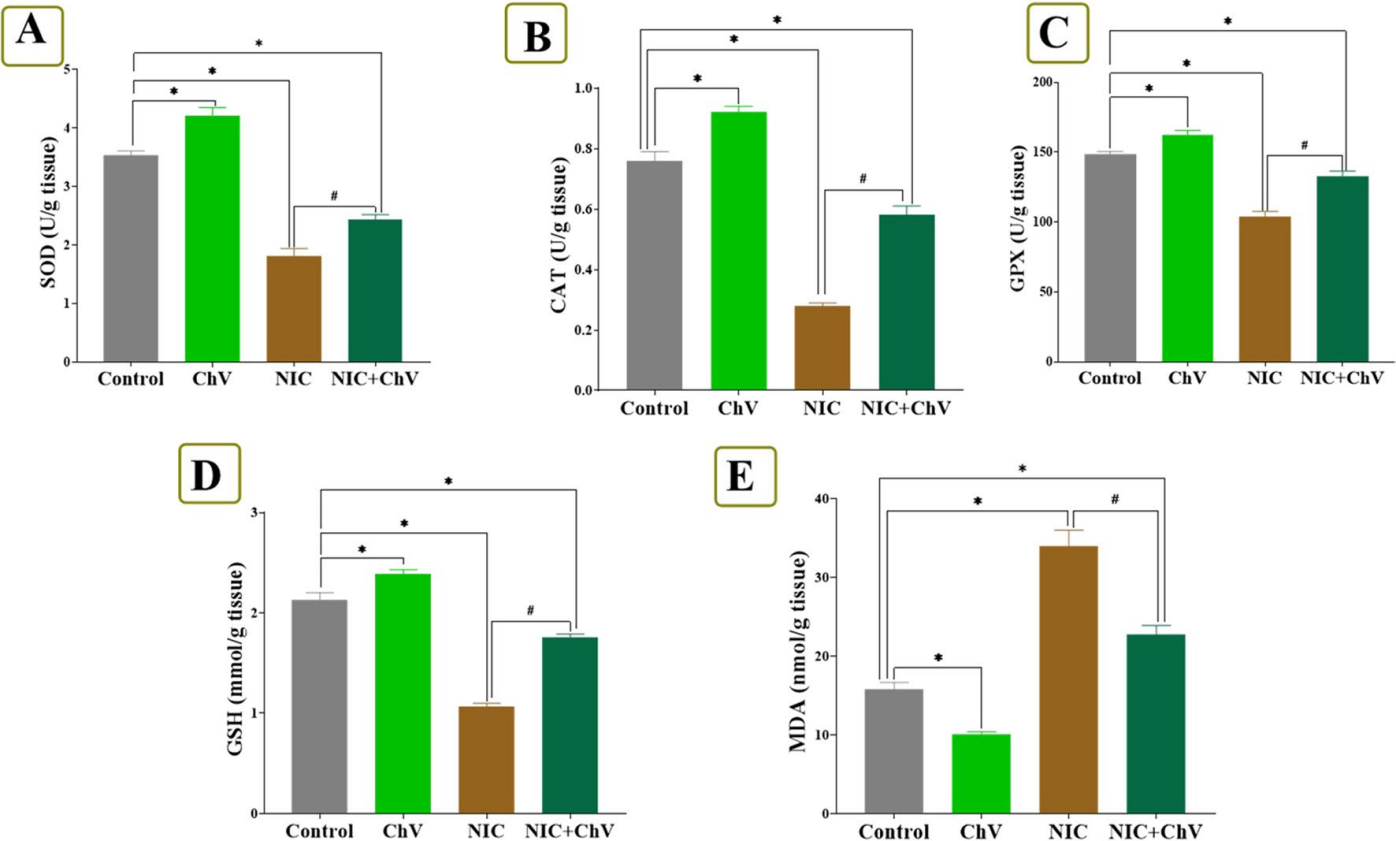
Effect of *Chlorella vulgaris* (ChV) and/or nicotine (NIC) exposure on hepatic oxidative stress and lipid peroxidation indices of Ehrlich ascites carcinoma (EAC) bearing female Swiss mice. **A** Superoxide dismutase (SOD). **B** Catalase (CAT). **C** Glutathione peroxidase 1 (GPX). **D** Reduced glutathione (GSH). **E** Malondialdehyde (MDA). Values are shown as mean ± SEM of 6 mice per experimental group. *P < 0.05 vs control, ^#^*P* < 0.05 vs NIC

**Fig. 2:**
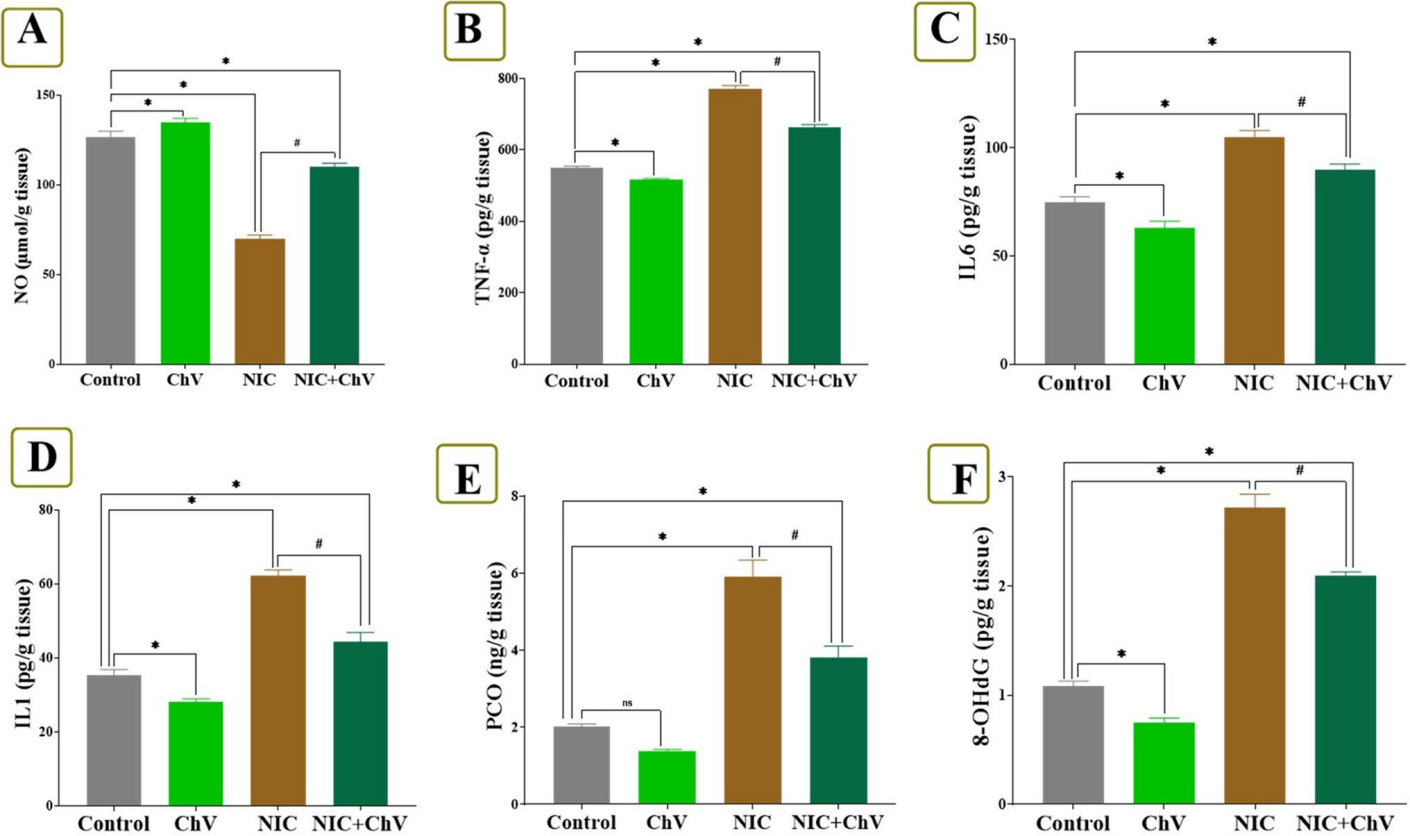
Effect of *Chlorella vulgaris* (ChV) and/or nicotine (NIC) exposure on hepatic inflammatory, DNA damage, and protein oxidative damage indices of Ehrlich ascites carcinoma (EAC) bearing female Swiss mice. **A** Nitric oxide (NO). **B** Tumor necrosis factor α (TNFα). **C** Interleukin-6 (IL-6). **D** Interleukin-1 (IL-1). **E** Protein carbonyl (PCO). **F** 8-hydroxy-deoxyguanosine (8-OHdG). Values are shown as mean ± SEM of 6 mice per experimental group. *P < 0.05 vs control, ^#^*P* < 0.05 vs NIC

The observed changes in oxidative stress and inflammation-related biomarkers in the liver of EAC-bearing Swiss female mice exposed to ChV and/or NIC are shown in Figs. 1 and 2. The ChV-treated group showed a significant decrease of NO, TNF-α, IL6, IL1, MDA, and 8-OHdG by 18.57%, 6.20%, 16.00%, 20.36%, 36.05%, and 30.56%, respectively, but exhibited a non-significant decrease by 31.53% in the PCO levels than the C group. ChV administration induced a significant increase in CAT, SOD, GSH, and GPx levels by 21.05%, 18.93%, 12.21%, and 9.07%, respectively, compared to the C group.

NIC exposure in the NIC group significantly increased levels of NO, TNF-α, IL6, IL1, MDA, PCO, and 8-OHdG by 45.28%, 39.83%, 39.47%, 77.28, onefold%, twofold, and twofold%, respectively, and produced a significant decrease in CAT, SOD, GSH, and GPX levels by 63.16%, 48.59%, 49.77%, and 30.12%, respectively, than the C group. Yet, ChV dosing in the NIC + ChV group significantly decreased the NIC-induced elevation of NO, TNF-α, IL6, IL1, MDA, PCO, and 8-OHdG hepatic levels to 24.83%, 20.34%, 20.00%, 26.08%, 44.69%, 78.19%, and 93.52%, respectively, than the C group. Also, ChV administration in the NIC + ChV group significantly restored the NIC-induced decrease of SOD, CAT, GSH, and GPX hepatic levels to 31.36%, 23.68%, 17.37%, and 10.64%, respectively, than the C group.

### Effect of ChV and/or NIC exposure combined with EAC on hepatic relative mRNA expression levels of oxidative stress-related genes

**Fig. 3:**
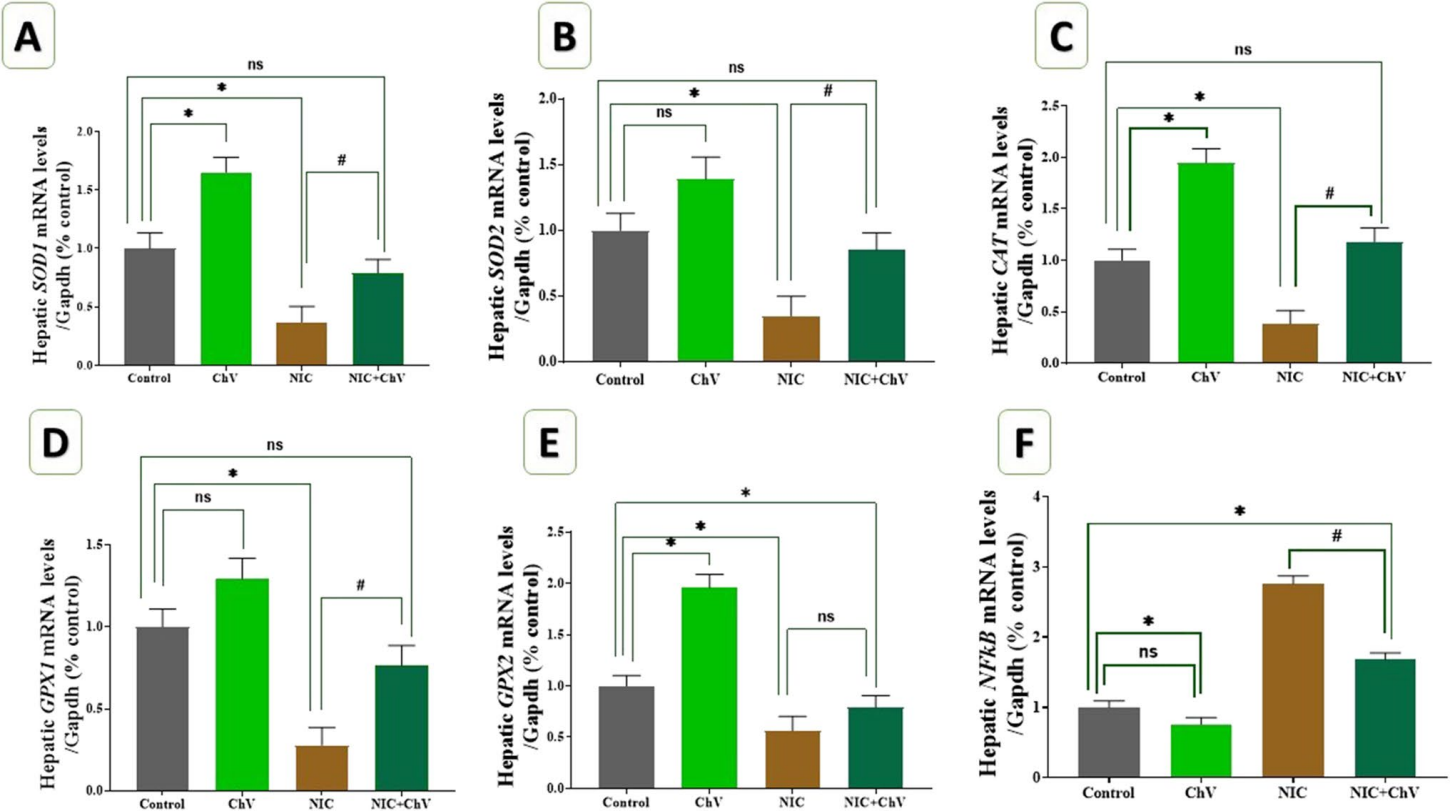
Effect of *Chlorella vulgaris* (ChV) and/or nicotine (NIC) on hepatic relative mRNA expression levels of SOD1, SOD2, CAT, GPx1, GPx2, and NF-κB of Ehrlich ascites carcinoma (EAC) bearing female Swiss mice. **A** Hepatic mRNA expression of superoxide dismutase 1 (SOD1). **B** Hepatic mRNA expression of superoxide dismutase 2 (SOD2). **C:** Hepatic mRNA expression of catalase (CAT). **D** Hepatic mRNA expression of glutathione peroxidase 1 (GPx1). **E** Hepatic mRNA expression of glutathione peroxidase 1 (GPx2). **F** Hepatic mRNA expression of nuclear factor kappa B (NF-κB). Values are shown as mean ± SEM of 3 mice per experimental group. *P < 0.05 vs control, ^#^*P* < 0.05 vs NIC

The effect of ChV and/or NIC exposure on hepatic relative mRNA expression levels of SOD2, SOD1, CAT, GPX2, GPX1, and NF-κB of EAC-bearing female Swiss mice is shown in Fig. 3. The mRNA levels of SOD2, SOD1, CAT, GPX2, and GPX1 were upregulated in the ChV administered group by 65.20%, 39.00%, 94%, 29.10%, and 95%, respectively, the upregulation was significant in the SOD1, CAT, and GPX2 genes expressions. In the meantime, there was a 24% decrease in NF-κB mRNA levels compared to the C group, which was not statistically significant.

The data showed that NIC exposure stimulated a significant downregulation of SOD1, SOD2, CAT, GPX1, and GPX2 expression levels by 62.80%, 65.10%, 61.40%, 72.2%, and 43.6%, respectively. On the contrary, mRNA levels of NF-κB were significantly upregulated by a twofold% increase in the NIC-exposed group if compared with the C group. ChV administration in the NIC + ChV group non-significantly modulated the NIC-induced downregulation of SOD1, SOD2, CAT, and GPX1 to 21.00%, 15.00%, 18.10%, and 24.00% reduction, respectively, while significantly modulated the GPX2 regulation by 21.00% reduction as compared with the C group. Additionally, ChV co-administration significantly triggered a significant modulation of the NIC up-regulated NF-κB mRNA expression levels in the NIC + ChV group to a 67.80% decline compared to the C group.

### Histopathological findings

**Fig. 4:**
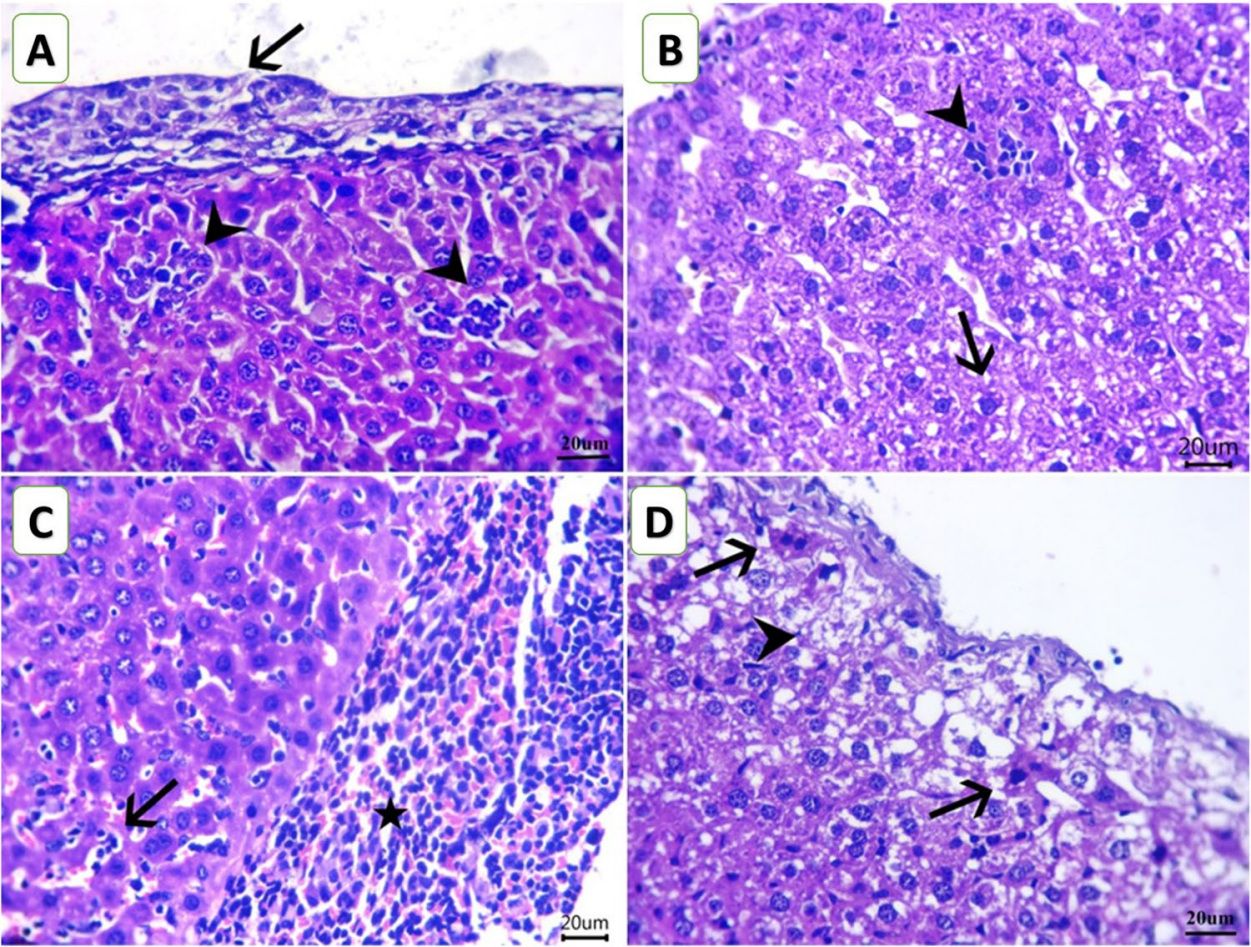
Photomicrograph of H&E-stained mice liver sections of mice from Ehrlich ascites carcinoma (EAC) bearing female Swiss mice of different groups (scale bar = 20 µm) showing** A** aggregates of Ehrlich ascites carcinoma cells adhered with hepatic capsule (arrow) and randomly distributed focal necrotic area invaded with leukocytic infiltrates (arrowheads) of the C group.** B** Hydropic degeneration (arrow) and scattered minute lymphocyte aggregates replaced the necrotic cells (arrowhead) of the ChV group. **C** Attached carcinoma cells to the hepatic capsule and admixed with intense hemorrhages and a marked number of lymphocytes (star) beside lymphocytosis within hepatic sinusoids (arrow) of the NIC group. **D** Intense accidental cell deaths (arrowhead) and programmed cell deaths (arrows), especially within subcapsular hepatocytes of the NIC + ChV group

Figure 4A–D shows serial sections from the livers of mice from all experimental groups. Microscopically, it is worth mentioning that the presence of the EAC cells outside the liver was a common finding in all mice groups, either in aggregates or as single cells resting in the liver capsule. The liver sections of the mice from the C group showed aggregates of EAC cells adhered to the hepatic capsule. The neoplastic cells had pleomorphic features with fine fibrin threads among them. Further, there are widely distributed areas of degenerative changes within hepatic cells besides randomly distributed focal necrotic areas that are invaded mostly with leukocytic infiltrates. The noticed inflammatory cells were mostly neutrophils and lymphocytes (Fig. 4A). The liver of mice from the ChV group exhibited few round cells within the hepatic capsule. In addition, degenerated hepatocytes, primarily hydropic and fatty degeneration, were seen. Moreover, scattered minute lymphocyte aggregates within sinusoids or replaced the necrotic cells were also noticed. Aggregates of EAC cells were observed above, but not attached to the liver capsule. Figure 4B displayed large areas of EAC cells attached to the hepatic capsule. The EAC cells were admixed with intense hemorrhages and a marked number of lymphocytes. The latter was also present within hepatic sinusoids of the NIC group with degenerative changes within most hepatic cells (Fig. 4C). The neoplastic cells were also seen inside the blood vessels and infiltrating the periportal triads, sometimes associated with leukocytic infiltration. The NIC + ChV group examined sections of liver tissue that declared a hyalinized hepatic capsule. Moreover, the subcapsular hepatic cells revealed intense degenerative changes that were mixed with accidental cell deaths and programmed cell deaths. However, few possible neoplastic cells were infrequently detected inside the blood vessels (Fig. 4D).

### Immunohistochemical findings

**Fig. 5:**
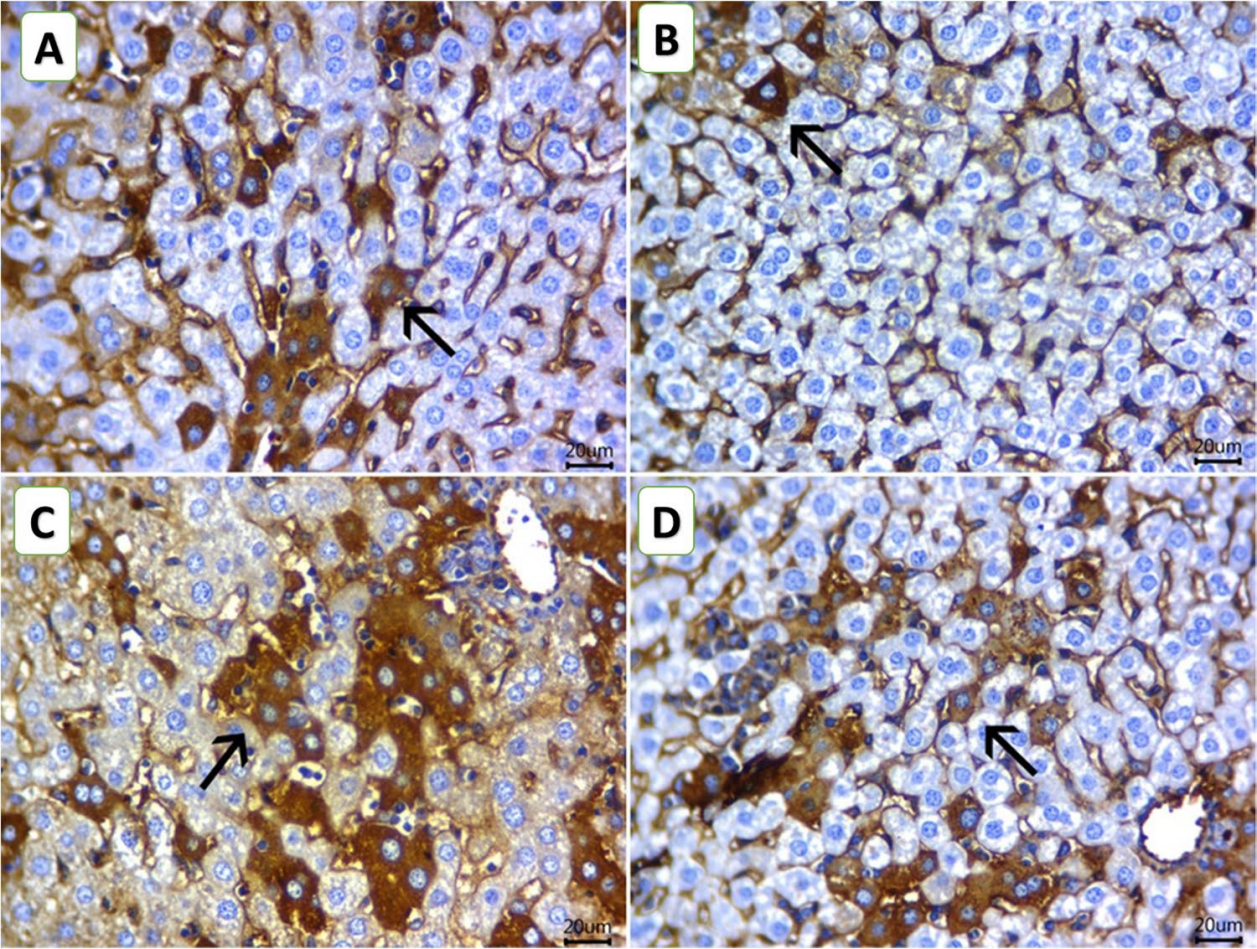
Representative photomicrograph for the hepatic immunostaining of caspase-3 (**A–D**) for the protective effect of *Chlorella vulgaris* (ChV) on nicotine (NIC) induced liver injury of Ehrlich ascites carcinoma (EAC) bearing female Swiss mice. **A** C group showing moderate cytoplasmic staining reaction for caspase-3 in hepatic tissue. **B** ChV group showing mild cytoplasmic staining reaction for caspase-3 in hepatic tissue. **C** NIC group showing strong cytoplasmic staining reactivity for caspase-3 in hepatic tissue. **D** NIC + ChV group showing a large number of mildly stained hepatocytes for caspase-3 in hepatic tissue. IHC counterstaining with Mayer’s hematoxylin. Arrows indicate positively stained cells (scale bar = 20 µm)

The scoring of the positive area of different immune-stained sections of hepatocytes is shown in Table 3. The immunostaining for caspase-3 is shown in Fig. 5A–D for different experimental groups. The examined liver sections of mice from the ChV group revealed mild cytoplasmic staining reactivity reaction for caspase-3% area of 5.33 ± 1.45 (Fig. 5B). On the contrary, examined liver sections of mice from the C and NIC groups revealed a significant increase of % area (12.00 ± 1.73 and 37.33 ± 2.60, respectively) of the hepatocytes exhibiting moderate to strong cytoplasmic staining reactivity for caspase-3 (Fig. 5 A and C). Examined liver sections of mice from the NIC + ChV group revealed that all the hepatocytes were mildly stained for caspase-3, % area (29.00 ± 3.78) (Fig. 5D).

**Table 3:** Scoring of the positive area of different immune-stained sections of hepatocytes with positive staining reactivity for caspase-3, Bax, and BcL2 in the liver of Ehrlich ascites carcinoma (EAC)-bearing female Swiss mice exposed to nicotine (NIC) and/or *Chlorella vulgaris* (ChV)

Markers	Groups			
	**C**	**ChV**	**NIC**	**NIC + ChV**
Caspase-3	12.00 ± 1.73	5.33 ± 1.45	37.33 ± 2.60 ^*^	29.00 ± 3.78 ^*^
Bax	14.66 ± 2.40	9.00 ± 1.73	42.00 ± 2.08 ^*^	27.00 ± 4.16 ^*#^
BcL2	10.00 ± 1.15	17.66 ± 2.33 ^*^	2.66 ± 0.88 ^*^	6.33 ± 1.76 ^#^

*Caspase-3*, cysteine aspartate specific protease-3; *Bax*, Bcl-2-associated X protein; *Bcl-2*, B-cell lymphoma-2. Values mean ± SEM of three animals per experimental group. Values mean ± SEM of three mice per experimental group. *P < 0.05 vs control, ^#^*P* < 0.05 vs NIC

**Fig. 6:**
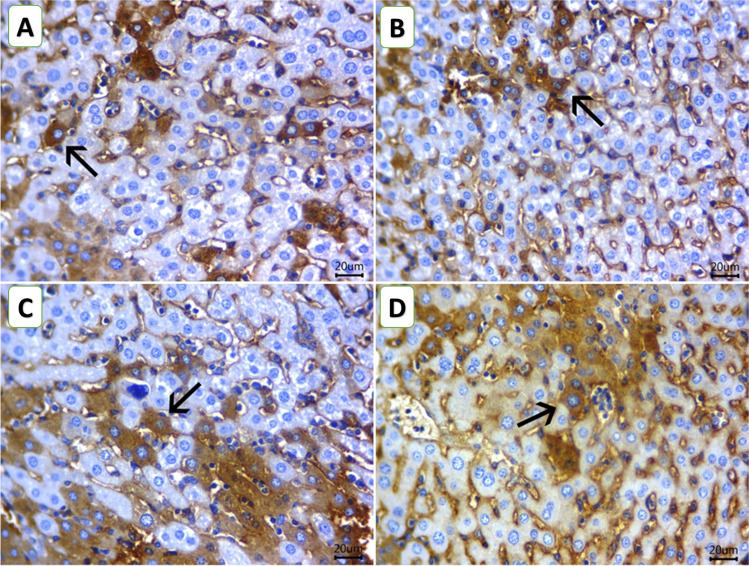
Representative photomicrograph for the hepatic immunostaining of Bax (**A**–**D**) for the protective effect of *Chlorella vulgaris* (ChV) on nicotine (NIC) induced liver injury of Ehrlich ascites carcinoma (EAC) bearing female Swiss mice. **A** C group showing moderate cytoplasmic labeling for Bax in hepatic tissue. **B** ChV group showing few positive cytoplasmic staining for Bax in hepatic tissue. **C** NIC group showing strong hepatic cytoplasmic labeling for Bax in hepatic tissue, **D** NIC + ChV group showing weak cytoplasmic labeling for Bax in hepatic tissue. IHC counterstaining with Mayer’s hematoxylin. Arrows indicate positively stained cells (scale bar = 20 µm)

Regarding immunostaining for Bax, as shown in Fig. 6A–D, examined liver sections of mice from the ChV group revealed few positive cytoplasmic staining for Bax in their hepatic tissue with a % area of 9.00 ± 1.73 (Fig. 6B). In contrast, examined liver sections of mice from the from the C and NIC groups revealed a significant increase of % area (14.66 ± 2.40 and 42.00 ± 2.08, respectively) of the hepatocytes exhibiting moderate to strong cytoplasmic reactivity for Bax (Fig. 6 A and C). Examined liver sections of mice from the NIC + ChV group revealed a significant decrease of % area (27.00 ± 4.16) of the hepatocytes exhibiting weak cytoplasmic reactivity for Bax (Fig. 6D).

**Fig. 7:**
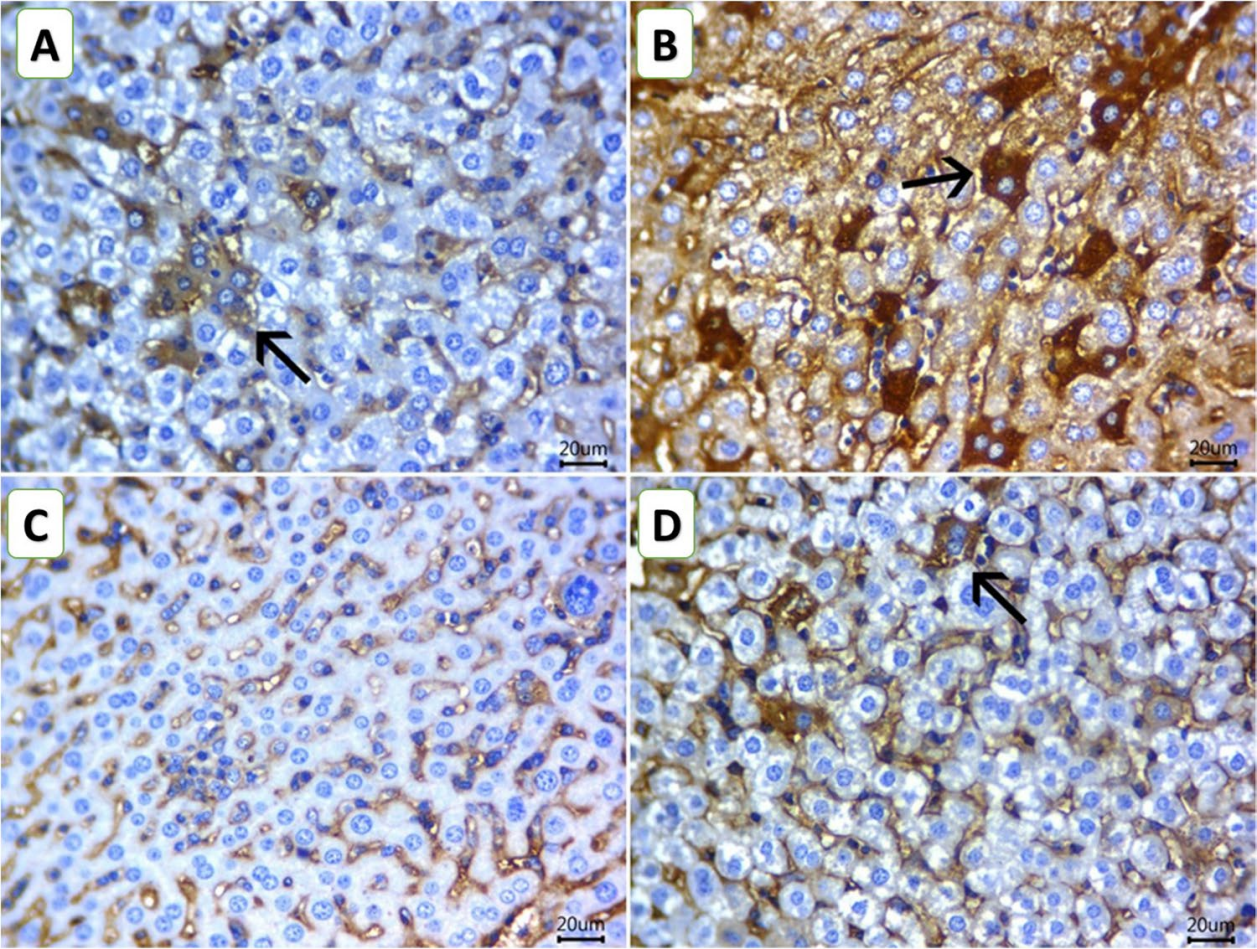
Representative photomicrograph for the hepatic Immunostaining of Bcl2 (**A**–**D**) for the protective effect of *Chlorella vulgaris* (ChV) on nicotine (NIC) induced liver injury of Ehrlich ascites carcinoma (EAC) bearing female Swiss mice. **A** C group showing mild cytoplasmic brownish staining reactivities for Bcl2 in hepatic tissue. **B** ChV group showing strong cytoplasmic brownish staining reactivities for Bcl2 in hepatic tissue. **C** NIC group showing very weak hepatic cytoplasmic staining reactivity for Bcl2 in hepatic tissue. **D** NIC + ChV group showing moderate cytoplasmic reactivity for Bcl2 in hepatic tissue. (Scale bar = 20 µm)

The immunostaining for Bcl2 is displayed in Fig. 7A–D. Examined liver sections of mice from the ChV group revealed hepatocytes with strong cytoplasmic brownish staining reactivities for Bcl2, with % area of 17.66 ± 2.33 (Fig. 7B). Examined liver sections of mice from the C and NIC groups revealed a significant decrease of % area (10.00 ± 1.15 and 2.66 ± 0.88, respectively) of the hepatocytes exhibiting mild to very weak cytoplasmic reactivity for Bcl2 (Fig. A and C). Examined liver sections of mice from the NIC + ChV group revealed that hepatocytes showed moderate cytoplasmic reactivity for Bcl2% area of 6.33 ± 1.76 (Fig. 7D).

## Discussion

The current study aimed to assess the ameliorative role of ChV administration against the detrimental effects induced by sub-chronic oral NIC exposure combined with EAC induction in female Swiss mice. The current research’s findings revealed that ChV significantly regulates the NIC-induced alterations in hepatic function, lipid profile, oxidative and inflammatory status, enhancing the mRNA expression pattern of antioxidant enzymes and pro-inflammatory cytokine-encoding genes and regulating the altered immune expression of caspase-3, Bax, and BcL2. EAC cells outside the liver were common in all mice groups, either in aggregates or as single cells resting on the liver capsule. Aggregates of EAC cells were observed above but not attached to the liver capsule in the ChV group, while multifocal expansion of hepatic sinusoids and inside the blood vessels by neoplastic cells was comparable to EAC cells detected outside the liver in the other mice groups. However, few possible neoplastic cells were infrequently detected inside the blood vessels with no evidence of considerable infiltration of EAC cells inside the sinusoids or in periportal areas in the NIC + ChV administered group, indicating the protective role of ChV.

Serum levels of AST, ALP, ALT, and LDH enzymes were considerably higher in the NIC group compared to the C group. Increased serum enzyme levels appear to reflect cellular leakage, structural damage, and membrane marker performance malfunction in the liver as a result of NIC treatment (Kolure et al. 2024). The observed abnormalities in NIC-treated mice could be explained by NIC-induced oxidative stress. Free radicals produced by NIC metabolism appear to attack polyunsaturated fatty acids and promote LPO. Also, they react with DNA and alkylating groups of membrane proteins and other cellular macromolecules, causing hepatic cell membrane damage, change in membrane permeability, and releases of hepatocyte cytozomal enzymes in serum. On the other hand, the serum levels of these enzymes are linked to liver function (Asante et al. 2016). Thus, free radicals appear to alter the enzymatic function and induce necrosis (Kolure et al. 2024). Moreover, the alterations in biochemical-related indices aligned with the liver histological injuries reported previously by Chen et al. (2016). It is also possible that it is due to hepatic damage caused by cancer cells invading the liver (Sannappa Gowda et al. 2022), which is evidenced in the current study by a multifocal expansion of hepatic sinusoids and inside the blood vessels by neoplastic cells comparable to EAC cells detected outside the liver in the NIC-exposed mice groups. On the contrary, liver function–related enzymes were reduced in the ChV-received groups; which could be suggestive of ChV’s protective effects on the liver because of its antioxidant activities and drop of oxidative stress induced by NIC toxicity. This was achieved by increasing the activity of enzymatic antioxidants and decreasing the hepatic CYP2E1 level. Our results are in line with those of Panahi et al. (2012a), who found that ChV treatment reduced AST, ALT, TG, and body weight in NAFLD patients. Additionally, Elsheikh et al. (2018) found that ChV extracts significantly reduced serum ALT activity by inhibiting deltamethrin-induced hepatotoxicity in rats.

The TC, TG, and LDL-C serum levels of the NIC-exposed group were significantly increased, but HDL-C was decreased considerably reflecting the dyslipidemic effect of NIC. In this respect, NIC augmented lipolysis in adipose tissue by combining with NIC-acetylcholine receptors (Andersson and Arner 2001), releasing free fatty acids. Consecutively, this resulted in an enhanced synthesis of TGs and VLDL-C in the liver. Moreover, the exposure of hepatocytes to generated free fatty acids and dyslipidemia resulted in the injury of mitochondria and ROS overproduction (Tian et al. 2023). Ateyya et al. (2017) reported that NIC has been shown to induce hepatic synthesis of TC, LDL-C, TGs, and VLDL-C, adversely affecting lipid profile, which matched with the current study findings. On the other hand, in the ChV-administered mice, a substantial improvement in the lipid profile was observed. Our results corroborate those of Li et al. (2013), who stated that ChV dosing decreased hepatic lipid accumulation from CCl_4_ exposure in mice. This may be attributed to hepatic inflammation and lipid metabolism modulation. Similarly, Cherng and Shih (2006) reported that ChV administration in high-fat diet–induced models corrected dyslipidemia via reducing TG, TC, and LDL cholesterol levels in the serum. ChV has been shown to have hypoglycemic and hypolipidemic effects (Barghchi et al. 2023). In rats, chlorella powder consumption resulted in a considerable reduction in serum and liver cholesterol and TG (Lee et al. 2009). Finally, Panahi et al. (2012b) established that 600 mg/day of ChV supplementation lowered TC, LDL-C, and TGs in dyslipidemic patients. TG lowering effects of ChV are possibly related to the lowering effect in plasma non-esterified fatty acid (NEFA) level because plasma NEFA is stored as TG in the liver (Barghchi et al. 2023).

Herein, NIC exposure decreased SOD, GPX, CAT, and GSH in liver tissues as well as increased LPO in the liver indicating oxidative stress in the NIC-exposed group. SOD is the first enzyme implicated in the detoxifying process and considered the most sensitive enzyme index in the hepatocellular injury. While CAT’s primary function is to scavenge H_2_O_2_ produced by free radicals or by SOD during the elimination of superoxide anions, it also converts H_2_O_2_ to H_2_O and O_2_. Because of its broad substrate requirements and great affinity for H_2_O_2_, the GPX is an effective ROS scavenger. Due to the antioxidant defense’s inefficiency in countering ROS-induced damage, these antioxidant enzymes are drastically reduced in the liver. The increased superoxide generation during NIC metabolism could explain the decrease in SOD activity in NIC-exposed animals (Kolure et al. 2024). The reduction of GPX activity in NIC-treated mice as obtained in this research indicated oxidative injury in the investigated tissues. Previous studies reported ROS production throughout NIC metabolism, such as hydroxyl radical, H_2_O_2_, superoxide anion, and NO (Hritcu et al. 2017). Therefore, the antioxidant enzyme inhibition in NIC-treated mice could promote LPO, alter gene expression, and lead to cell death.

Herein, hepatic NO level significantly increases in NIC-exposed mice, while ChV decreases NO. Moreover, the absorption of NIC in the body is accompanied by the triggering of serum NO and oxidative stress levels (Lallemand et al. 2006). Similarly, Khaled et al. (2020) showed that NIC enhanced iNOS protein levels in lung and liver tissues, implying that iNOS mediated the high amounts of NO. Interestingly, in the previous study, the ChV supplementation resulted in a hepatoprotective effect through suppressing the hepatic oxidative stress. In CCl_4_-induced acute liver damage, Li et al. (2013) found that treating animals with ChV daily for 4 weeks significantly reduced MDA levels. Surprisingly, compared to the control group, the ChV group showed a decrease in total oxidative stress and MDA levels and an increase in TAC. Consequently, under typical circumstances, the oxidative stress status decreased after ChV feeding. Additionally, ChV extracts raised GPx, CAT, and SOD activity in liver tissue while decreasing hepatic MDA and NO levels. Consistent with our results, Elsheikh et al. (2018) observed that administering 50 mg/kg BW of ChV powder to rats for 2 months raised CAT and SOD activity and decreased MDA levels. These results could be attributable to the antioxidants included in the ChV extract, including ascorbic acid, α and b carotenes, tocopherol, and lutein (Mohamed et al. 2022). Furthermore, ChV can directly and indirectly reduce free radical-induced damage via scavenging free radicals or stimulating antioxidant enzyme activity (Abdel-Khalek et al. 2023). ChV has chlorophylls, which reduce ROS production and hence prevent LPO (Pérez-Gálvez et al. 2020). Furthermore, ChV exhibits strong scavenging action for superoxide anion radicals and NO owing to its phytochemical compounds like carotenoids and terpenoids (Safafar et al. 2015). Similarly, ChV protective and antioxidant effects are related to their content of phenolic compounds, which play a crucial role in free radicals scavenging, oxygen inhibition, and peroxide decomposition (Martins et al. 2016; Renugadevi et al. 2018). Additionally, Abu-Serie et al. (2018) found that ChV extracts significantly reduced LPO and TBARS amounts in leukocytes.

Smoking induces oxidative stress linked with LPO, leading to stellate cell activation and fibrosis. Smoking also boosts the pro-inflammatory cytokines (IL-1, IL-6, and TNF-α) generation, which has been linked to pathophysiological problems, including organ damage (Andersen et al. 2021). TNF-α is a well-known factor that causes liver injury (Chen and Ma 2019). As a result, the higher serum levels of pro-inflammatory cytokines IL-1 and TNF-α in NIC-exposed animals could elucidate the histological changes in hepatic tissue and raised liver function enzyme activity seen in this group. The current study suggests that the increased oxidative stress might influence the elevated levels of pro-inflammatory cytokines, such as TNF-α and IL-1β (Elsherbiny et al. 2017). Measured levels of NFκB in liver homogenates by Khaled et al. (2020) have reported that NIC-induced tissue inflammation primarily by boosted NF-κB and TNF-α cytokine expression. It has been conveyed that NIC initiates α7 nicotinic acetylcholine receptors on macrophages, which in turn initiate the pro-inflammatory mediators, resulting in the production of free radicals and inflammatory cytokines that are involved in systemic inflammation and tissue damage (Zahran and Emam 2018). NFκB is a crucial transcription factor that stimulates a great gene number accused in inflammation, like TNF-α (Boisson et al. 2020). NIC has been shown to increase NF-κB protein levels in liver, lung, and kidney tissues (Cooper and Magwere 2008; Martins et al. 2016; Zahran and Emam 2018).

According to the findings of this study, giving rodents the ChV caused a drop in TNF-α and IL1-β levels. ChV’s anti-inflammatory properties could explain why it protects against NIC-induced liver damage. This finding is consistent with Abu-Serie et al. (2018), who suggested that ChV’s anti-inflammatory action could be attributed to specific phenolic compounds such as gallates, which are powerful NO and TNF inhibitors. Finally, ChV extract reduced the pathological lesions caused by nitrite in hepatic tissues. These findings resembled those of Abd-Elmoneim and Darwish (2016), who found that giving mice an aqueous extract of ChV at a concentration of 500 mg/kg BW for 28 days improved antioxidant activities and reduced LPO, resulting in hepato-protection against monosodium glutamate.

The toxic effects of NIC are attributable, at least in part, to an increase in the generation of free radicals and ROS (Kolure et al. 2024). Another known mechanism by which NIC damages the system is by inducing inflammation. NF-κB activates over 200 genes that have been found to decrease apoptosis induce cell transformation, invasion, metastasis, chemo-resistance, and radio-resistance (Boisson et al. 2020). Smokeless tobacco extract promotes the expression of NF-κB in oral premalignant and cancer cells (Li et al. 2018). The specific mechanism by which NIC induces NF-κB is unknown. However, previous reports showed that NIC, even at the physiological level of 0.8 µM, increases oxidative stress and the redox-sensitive transcription factor, NF-κB (Crowley-Weber et al. 2003). As far as we know, there is no published data regarding the NIC-induced alterations in the antioxidants and inflammation‐related gene expression levels in different tissues such as the liver, kidneys, and lungs. Our findings, based on an RT-PCR gene expression approach, validated the findings of Ivey et al. (2014), which indicated the involvement of caspase-2 and iNOS-mediated apoptotic pathway in NIC plus high-fat diet–induced hepatocellular apoptosis. They discovered a considerable increase in the amount of active caspase-2 protein. El-Sherbeeny et al. (2016) found that NIC treatment boosted caspase-3 activity and lowered NO levels in liver cells. Furthermore, the presence of NIC created OS, which damaged the arteries and caused inflammation in the liver. NIC and cotinine have been proven to cause apoptosis in the liver either directly or immunologically. NIC-induced ROS generation can enhance caspase-2 activity as well as NO synthesis. NO production, on the other hand, has been linked to cell death and damage (Yeo et al. 2022). NIC can encourage an existing tumor that was started by other variables through its genotoxic effects, as well as by aiding tumor cell survival, growth, metastasis, and resistance to chemotherapy (Grando 2014). NIC can thus be pro or anti-apoptotic, depending on the quantity of the drug, species-specific changes in NIC metabolism, and the target cells.

Members of the Bcl-2 family, pro-apoptotic proteins (Bak, Bid, Bax), and anti-apoptotic proteins (Bcl-XL, Bcl-2) strictly control the balance between cell life and cell death. By binding to Bax, Bcl-2 shields cells against apoptosis prevents oligomerization and translocation to mitochondria, triggers permeability transition, and, as a result, activates the caspase cascade (Elkon and Oberst 2025). Different groups have investigated the unique apoptotic effects of NIC in vitro and in vivo, finding a link between NIC exposure and apoptosis. NIC has been reported to induce apoptosis in some earlier reports (Galitovsky et al. 2004; Zhao and Reece 2005), whereas others found that NIC prevents apoptosis (Wielgus et al. 2004; Copeland et al. 2005; Zhao and Reece 2005). The current investigation examined the apoptotic effect of NIC exposure on the liver of female Swiss mice in light of the conflicting data regarding the consequences of NIC toxicity. The results of real-time PCR demonstrated that the hazardous dose of NIC induced apoptosis in the liver via the caspase-3 apoptotic pathway. Because the liver is the key site of NIC metabolism, apoptosis is triggered in the liver. Assuming that the liver metabolizes the majority of NIC, each transit through the liver extracts roughly 70% of the drug from the blood. Furthermore, ChV treatment reduced caspase-3 activation in hepatic tissues, according to our findings. This could be because of ChV’s antioxidant properties. This outcome is consistent with past research findings (Saberbaghi et al. 2013; Abd-Elmoneim and Darwish 2016).

Our study has some limitations that should be considered when interpreting the results. Although our study examined various hepatic function indices, gene expressions, and immunostaining, further studies, such as molecular investigations or pathway analyses, are necessary to elucidate the other probable mechanisms involved in NIC-induced hepatic injury and the ameliorative effects of ChV extract. Moreover, while our findings suggest the potential benefits of ChV extract in mitigating NIC-induced hepatic injury, it is important to note that additional research is needed to determine the therapeutic efficacy and safety of ChV in human cancer patients. Clinical trials and further preclinical studies are required to validate our findings and establish appropriate dosage regimens for human use.

## Conclusion

The current study found that ChV protects female Swiss mice livers from the harmful effects of sub-chronic oral NIC exposure combined with EAC induction by regulating NIC-induced changes in hepatic function, lipid profile, oxidative and inflammatory injury, enhancing the mRNA expression pattern of antioxidant enzymes, and pro-inflammatory cytokine-encoding genes, and regulating the altered immunostaining of caspase-3, Bax, and BcL2. Furthermore, ChV protects against EAC cell metastasis, with no indication of significant EAC cell infiltration in the sinusoids or periportal areas of the liver. Overall, the findings of this work highly recommended further human studies to assess the efficacy of ChV in its administration to cancer patients who are consuming NIC daily, whether from smoking or exposure to secondhand smoke.

## Data Availability

Data is provided within the manuscript or supplementary information files.

## Abbreviations

NIC: Nicotine
ChV: *Chlorella vulgaris*
ROS: Reactive oxygen species
EAC: Ehrlich ascites carcinoma
TNF-α: Tumor necrosis factors
*NF-κB*: Nuclear factor-kappa B
NRT: Nicotine replacement therapy
LPO: Lipid peroxidation
Caspase-3: Cysteine aspartate specific protease-3
Bax: Bcl-2-associated X protein
Bcl-2: B-cell lymphoma-2
MDA: Malondialdehyde
PCO: Protein carbonyl
8OH2dG: 8-Hydroxy-2-deoxyguanosine
*GAPDH*: Glyceraldehyde-3-phosphate dehydrogenase
*SOD1*: Superoxide dismutase 1
*SOD2*: Superoxide dismutase 2
*CAT*: Catalase
*GPX1*: Glutathione peroxidase 1
*GPX2*: Glutathione peroxidase 2
